# AToMS: A Ubiquitous Teleconsultation System for Supporting AMI Patients with Prehospital Thrombolysis

**DOI:** 10.1155/2011/560209

**Published:** 2011-07-06

**Authors:** Bruno S. P. M. Correa, Bernardo Gonçalves, Iuri M. Teixeira, Antônio T. A. Gomes, Artur Ziviani

**Affiliations:** National Laboratory for Scientific Computing (LNCC), National Institute of Science and Technology on Medicine Assisted by Scientific Computing (INCT-MACC), 25651-075 Petrópolis, RJ, Brazil

## Abstract

The latest population-based studies in the medical literature worldwide indicate that acute myocardial infarction (AMI) patients still experience prolonged delay to be rescued, which often results in morbidity and mortality. This paper reports from a technological standpoint a teleconsultation and monitoring system named AToMS. This system addresses the problem of prehospital delivery of thrombolysis to AMI patients by enabling the remote interaction of the paramedics and a cardiologist available at a Coronary Care Unit (CCU). Such interaction allows the diagnosis of the patient eligibility to the immediate application of thrombolysis, which is meant to reduce the delay between the onset of symptoms and the eventual application of proper treatment. Such delay reduction is meant to increase the AMI patient's chances of survival and decrease the risks of postinfarction sequels. The teleconsultation is held with the support of wireless and mobile technologies, which also allows the cardiologist to monitor the patient while he/she is being taken to the nearest CCU. All exchanged messages among paramedics and cardiologists are recorded to render an auditable system. AToMS has been deployed in a first stage in the city of Rio de Janeiro, where the medical team involved in the project has conducted commissioned tests.

## 1. Introduction

Acute myocardial infarction (AMI) is among the leading causes of death and physical incapacity worldwide [1]. In 2005, for instance, cardiovascular diseases accounted for 30% of deaths globally [2]. It is also known that morbidity and mortality of patients experiencing AMI may be lessened by decreasing the interval from symptom onset and its recognition to treatment [3]. Nevertheless, in both industrialized and developing countries the so-called ST-segment elevation myocardial infarction (STEMI) programs—which depend on delivering earlier (typically prehospital) emergency medical services (EMS)—are still being studied and revised in order to be efficiently implemented [4–7]. As a result, AMI patients still experience prolonged delays to be provided with initial medical care [8–10].

Among the causes of AMI (which is in general STEMI), the most common is ischemia, which is a sudden reduction or interruption of the blood flow to a tissue because of an arterial constriction or obstruction. To date, there are two main treatments in cardiology to resume the blood flow in a previously constricted or obstructed artery: *angioplasty* and *thrombolysis* (we discuss both in more detail in Section 2.1). Crucially, the latter has the benefits over the former of being cheaper and feasible to be implemented by the paramedic him/herself at the emergency scenario. Nevertheless, the call for the application of thrombolysis requires a careful technical judgement on whether or not the AMI patient is thrombolytic eligible. Such a judgement often can only be made by a cardiologist. This specialized decision is based on an analysis of an (usually ten-second) electrocardiogram (ECG) in addition to some information on the recent medical history of the patient. Here is where our problem arises. As a matter of fact, such a specialist is most likely not available at the place where the first EMS is provided to the patient, thus rendering rates of prehospital thrombolytic administration lower than recommended by clinical guidelines. 

In this paper, we report a teleconsultation system that enables the remote interaction of paramedics who are providing the AMI patient with prehospital EMS and a cardiologist available at a Coronary Care Unit (CCU). The focus of the paper is on a comprehensive description of how the system addresses the medical problem aforementioned from a technological point of view.

The system is named AToMS, standing for AMI Teleconsultation & Monitoring System. AToMS takes advantage of ubiquitous (mobile and wireless) technologies to be an effective telemedicine resource anytime, anywhere. Nevertheless, as we discuss in Section 5.1, connectivity and bandwidth limitations in Brazil, and also a preference for using structured data—easier to store and retrieve in view of auditability, a fundamental concern for the delivery of prehospital thrombolysis—have also been considered in the adoption of technologies.

The remainder of this paper is organized as follows. In Section 2, we briefly discuss thrombolysis and angioplasty, which are the most adopted reperfusion therapies for AMI patients. We then introduce the particular scenario of Brazil regarding the provision of EMS for AMI patients. We describe the AToMS system in Section 3, presenting a system overview by focusing on the communication flow and the architectural components. Subsequently, in Section 4, we elaborate on the system implementation details. We then proceed to comment in Section 5 on related work on telemedicine systems dedicated to the problem of supporting AMI patients with EMS and/or prehospital ECG monitoring. We also discuss in this section: (i) the requirement for auditability; (ii) AToMS' limitations and the future work we have delineated; (iii) the system applicability, deployment, and socioeconomic perspectives. To conclude the paper, we provide in Section 6 a summary of the key contributions conveyed here as well as our final considerations.

## 2. Background

### 2.1. Thrombolysis versus Primary Angioplasty

*Thrombolysis* therapy (also called fibrinolysis) consists in administering thrombolytic drugs that dissolve the arterial obstruction, so that the blood flow to the patient's heart muscle can be restored effectively. *Angioplasty*, or *primary percutaneous coronary intervention* (PPCI), is a more expensive though highly-adopted procedure in ischemic AMI cases that is based on a surgical intervention to unblock the obstructed artery.

In the medical literature (e.g., [3, 11–13]), there is a long-running discussion on which reperfusion therapy—thrombolysis or angioplasty—is the most suitable one. In this comparison, arguments for both thrombolysis and angioplasty have been claimed. To quote Stone [11], *“[o]ne of the longest running debates in cardiology has concerned the best reperfusion therapy in patients with evolving acute myocardial infarction (AMI).”* According to the current guidelines [13], PPCI is the preferred form of reperfusion treatment for patients with STEMI. However, there is consensus that (i) reducing the time from first contact with the health care system to the initiation of reperfusion therapy (the so-called *system delay*) is key [10]; (ii) early administration of prehospital thrombolysis (PHT) is an alternative to PPCI that may produce similar outcomes at a reduced cost [14]; (iii) if the patient is thrombolytic eligible, then the combination of PHT delivery with PPCI as a secondary intervention after hospital admission achieves prominent results [2]—for example, Arntz [15, p. S93] reports on a study where TIMI grade III flow (TIMI stands for “Thrombolysis In Myocardial Infarction” and is the name of a study group coordinating several trials, particularly focusing on percutaneous coronary intervention, thrombolysis, and cardiovascular disease in general. TIMI grade III flow means complete perfusion, that is, a normal flow has been restored to fill the distal coronary bed completely. For information on the meaning of each grade, please refer to http://www.timi.org/?page_id=76.) was achieved in 91% of patients without major complications. Furthermore, studies comprising actual telemedicine systems implementing the delivery of PHT and suggesting it is feasible also exist (see, for instance, [16]).

Inline with this trend, we resume this section by referring to Danchin et al.'s conclusion which has been a result from their review recently published on this matter [3].

 “*Although PPCI is the treatment of choice, it is often not possible to implement it within the required time window. […] In areas where PPCI is not (immediately) available, thrombolysis remains the only treatment option and should be administered as soon as possible, preferably prehospital.”*

We adhere to it in the work reported hereon, by also considering the particular context of Brazil and its current guidelines as discussed in the next section.

### 2.2. Prehospital Thrombolysis for AMI Patients in Brazil

Many developing countries, such as Brazil, have large socioeconomic contrasts characterized by a deep social inequality and a strongly imbalanced distribution of the produced wealth. Concerning healthcare, telemedicine diffusion may significantly help narrowing these gaps in developing countries [17, 18]. In Brazil, in addition to a call for PHT delivery in the cardiology research literature [19], recent federal policies have been proposing “thoracic pain protocols” combining the use of both thrombolysis and PPCI in AMI cases. The main goal of such protocols is to reduce the system delay in AMI cases by increasing the adoption of PHT by emergency teams. At the regional level, health authorities in Rio de Janeiro have established a project called TIET [20] (standing for “Thrombolytic treatment of AMI on emergency with teleconsultation”). Essentially, the TIET project aims at the following:

- displacing the thrombolytic treatment from CCUs in large hospitals to prehospital care facilities, which receive patients rescued by emergency teams;
- providing a teleconsultation infrastructure that offers means for cardiologists to assist emergency teams in deciding upon the eligibility of a given AMI patient for thrombolytic treatment.

As of March 2005, a resolution from the public health authorities in Rio de Janeiro establishes the deployment of the TIET project in all its public health emergency units. One of the requirements defined in this project is that these emergency units should have electrocardiographs and fax machines connected to conventional phonelines. The main goal of this requirement is to provide a sort of rudimentary teleconsultation infrastructure by allowing a paramedic to confirm with a cardiologist the AMI diagnosis and the eligibility of the patient for PHT when ECGs are not clear enough or the paramedic is not experienced enough. Furthermore, if the AMI case so demands, this infrastructure provides the CCU with earlier patient information to prepare it for his/her imminent arrival.

The TIET project, as initially conceived, presents two main limitations on its effectiveness and efficiency from a technological standpoint:

- the TIET teleconsultation system is hard to audit. As there is no centralized record in the original TIET teleconsultation system, direct auditing actions become particularly difficult to be successfully implemented, thus limiting the enforcement of PHT delivery in the indicated cases—we return to this point in Section 5.2;
- the TIET teleconsultation system relies on a fixed communication infrastructure, forcing the AMI patient to be displaced to an emergency unit before being properly treated with thrombolytics. Depending on how difficult the access is to the closest emergency unit, the thrombolytic administration may be made unfeasible to this patient due to the accumulated system delay.

The medical and public policies were already set, however, for upgrading the TIET project with the available mobile and wireless technologies such as GPRS/EDGE/3G and Web services (SOAP/XML). As put by Hung and Zhang [21], these technologies play a key role for enabling telemedicine initiatives with longer reach and easier deployment. The objective of supporting AMI cases efficiently with PHT delivery thus gave rise to AToMS, the teleconsultation system introduced next which is meant to deal with the TIET shortcomings.

## 3. The AToMS System

The design and implementation of AToMS relies on wireless and mobile communications to assist paramedics (emergency team) and cardiologists to

- exchange information about an AMI patient and decide on its eligibility for receiving thrombolytics in a timely fashion;
- exchange complementary information about the patient's condition (including additional ECGs) while the he/she is being displaced to a CCU.

The AToMS system aims at covering not only conventional emergency units, but virtually *any* place enabled with a wireless network where the AMI patient can first be approached by a mobile emergency team or ambulance service. To achieve this goal, the system makes use of different networking technologies to enhance its ubiquitousness. Human intervention in AToMS is usually restricted to requests for consultation from paramedics and replies to such a consultation from cardiologists. A request for consultation is made by sending an *analysis request* from the AMI patient that comprises one or more digitalized ECG records and clinical information just collected by the paramedics from the patient. Additional patient information might be retrieved from external sources by the system coordination server (Although this feature is not implemented yet, it is worthwhile to highlight that it turns out to be one of the advantages of having a central coordinator in the system architecture). The system then conveys the patient analysis request to a cardiologist, who can then take proper decisions on whether or not the patient is thrombolytic eligible earlier in the prehospital setting.

### 3.1. System Overview and Architecture

**Figure 1:**
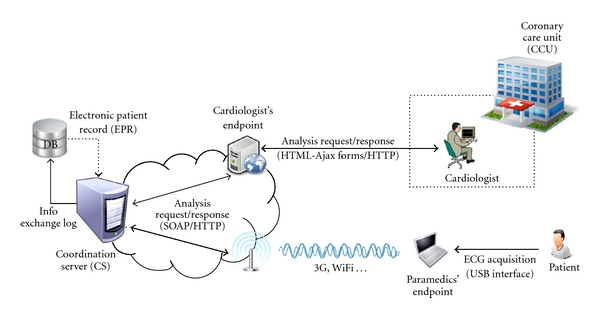
AToMS' system overview.

Figure 1 provides an overview of the AToMS communication flow, structured according to the client-server architectural style.

The coordination server (CS) is concerned with the information exchange between paramedics and cardiologists. The CS receives *analysis requests* from paramedics at one client side and forwards them to a cardiologist logged on the system at the other client side. By doing that, the CS stores all information exchange in a database for enabling further auditability (cf. Section 5.2).

The client side is composed of two different applications—which configure our two endpoints—used by the paramedics on one side, and cardiologists on the other side. The paramedics use an application that runs on top of a 3G-capable laptop computer connected through a USB interface to a proprietary ECG acquiring device (The particular hardware setting that constitutes the system prototype is described in Section 4.1. Notice, nonetheless, that the architectural design is not restricted to specific wireless and mobile technologies.). This application allows them to

- fill in an electronic patient record (EPR) (By EPR we do not mean a complete and permanent electronic health record (EHR) of the patient, but a local record lying in the patient clinical information for purposes of the telediagnosis by the cardiologist.) with (a) the patient's clinical information—for example, heart rate, information for thrombolysis exclusion criteria, and so forth—and (b) the patient's medical history just collected by anamnesis—for example, history for angioplasty, hypertension, diabetes, and so forth;
- acquire from the patient an (12-lead or less) ECG record (codified by the ECG acquiring device as a file) to be added to the EPR just mentioned;
- transmit all this data through a wireless (e.g., 3G) connection, thus defining the issue of an *analysis request*, possibly followed by chat messages to an available cardiologist.

The CS then forwards the analysis request and the chat messages to the cardiologist available on the system at the nearest Coronary Care Unit (CCU). (The system allows for different CCUs being involved).

Notice that at least one cardiologist must be logged on AToMS per CCU (The medical team of the AToMS project responds for this requirement.), otherwise the teleconsultation has no purpose. The cardiologist's endpoint consists of a web application that runs on top of a standard web browser. The application receives from the CS a list of pending analysis requests which permits the cardiologist to answer multiple requests simultaneously. Each analysis request is transmitted by the CS to the cardiologist by HTTP as he/she logs into the system and then exhibited to him/her as an HTML webpage. This page lies in the following:

- both the EPR and download link(s) to the ECG record(s) collected by the paramedics for the cardiologist to assess;
- a web form that molds the medical report the cardiologist is to assign to the request. This includes marking whether or not the thrombolytics should be ministered to the patient as well as the severity of the patient's condition according to his/her technical judgement;
- a chat channel to allow real-time communication between the paramedics and the attending cardiologist.

Every medical report (i.e., the web form filled in) assigned by a cardiologist is sent to the CS, which in turn passes it by to the paramedics. Additional ECG records together with the chat channel resource allow for the cardiologist to get from the paramedics the patient's actual condition while he/she is being taken to the CCU. This provides the CCU's team with actual patient information from the prehospital setting, so that they can get prepared for the subsequent proper treatment to that particular patient at the CCU.

The paramedics' laptop computer must be enabled with wireless technologies (e.g., GPRS/EDGE/3G, Wi-Fi, etc.) for connectivity with the CS. We describe in Section 4 the technology used to address this and all the other requirements involved in the communication carried out between the CS and both of the client-side applications—that is, transmission of EPR, clinical reports, ECG records, and chat messages.

### 3.2. Aspect-Oriented Programming

Nonfunctional requirements have been considered since the architectural design specification by using the so-called aspect-oriented programming (AOP) approach [22]. This programming paradigm consists in an attempt to increase modularity by allowing the separation of code that handles cross-cutting concerns. For example, let us consider the security concern. It defies usual encapsulation programming abstractions (e.g., procedures, modules, classes, and methods) because it “cuts across” many of them in a program. Aspect-oriented programming entails handling a concern such as security apart from the main program logic for the sake of modularity.

**Figure 2:**
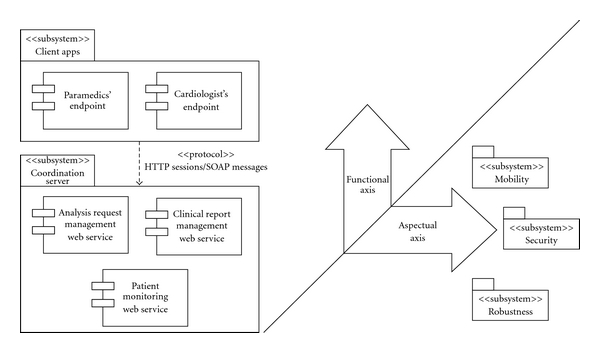
Functional and aspectual axes in the AToMS system.

AOP has been employed in the AToMS' architecture in order to manage concerns that, otherwise, would be introduced into the system intrusively (i.e., mixed up within the system functionalities). We have adopted an architectural vision sectioned in two axes, namely, the functional and the aspectual axes (see Figure 2). The *functional axis* comprises, as the name suggests, the application functional requirements—namely, managing analysis requests and clinical reports, and allowing patient monitoring. The aspectual axis in turn cross-cuts the functional axis vertically in order to meet the system nonfunctional requirements, namely,

- *security*: the inherent nature of medical data requires confidentiality;
- *robustness*: that is, the system must be reliable and available;
- *mobility*: not only should the system take advantage of mobile and wireless technologies, but also guarantee mobility and roaming by handling their heterogeneity (e.g., Wi-Fi, WiMax, GPRS/EDGE/3G, etc.).

For the time being, we have met the security issue as described in the implementation section (see Section 4.5). We aim at meeting the two other nonfunctional requirements by using aspect-oriented programming as well in future work, as discussed in Section 5.5.

## 4. Implementation Details

We describe in this section implementation details of the AToMS' software components and also the hardware employed in the system.

### 4.1. Hardware Devices

**Figure 3:**
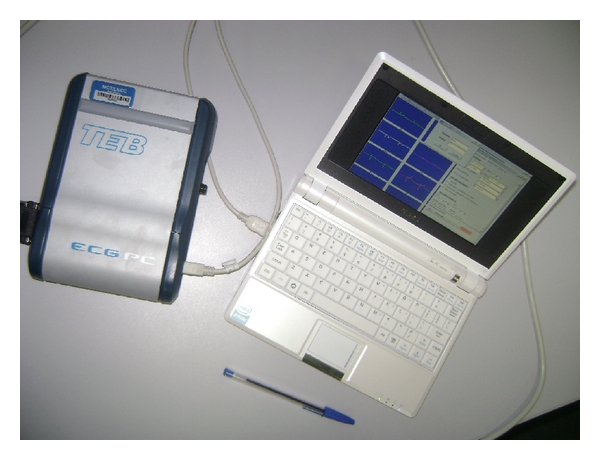
Hardware equipment used by the paramedics.

In order to firstly assist the AMI patient at the emergency scenario, the paramedics make use of an ANVISA-approved (ANVISA is the Brazilian agency for the regulation of Health-related products (i.e., similar to FDA in US)) ECG acquiring device featured with a USB port for interfacing with a laptop or mobile device. We adopted this setting because there is currently no ANVISA-approved ECG acquiring device featured with short-range wireless capabilities such as Bluetooth or ZigBee interfaces. This ECG device is connected to a 3G-capable, highly-portable laptop computer featuring a 900 MHz processor, 512 Mb memory, and a 7′′ display screen (max. resolution of 800 × 400 pixels); see Figure 3.

### 4.2. Coordination Server

The CS has been developed as a set of web services implemented in Java using the API JAX-WS. Currently, this set of web services comprise the following:

- RequestWS: meant for the paramedics to send the analysis requests to the cardiologists;
- RegisterWS: for the paramedics to send to the CS additional patient (personal) information—collected *after* sending the critical clinical information—which is integrated into the very same EPR of the patient;
- ResponseWS: for the cardiologists to send their clinical report to the paramedics;
- ChatWS: for paramedics and cardiologists to be able to exchange chat messages. This feature also supports the transmission of additional ECG records for the sake of keeping the physician posted and/or allowing him/her to conduct a deeper investigation as the patient is receiving EMS.

All the data just described—namely, the analysis request, ECG records, chat messages, and clinical reports assigned by the cardiologist—is safely stored in a *PostgreSQL* database system. These data is coded into Java classes which are mapped into relational tables by using the Hibernate framework.

### 4.3. Paramedics' Endpoint

**Figure 4:**
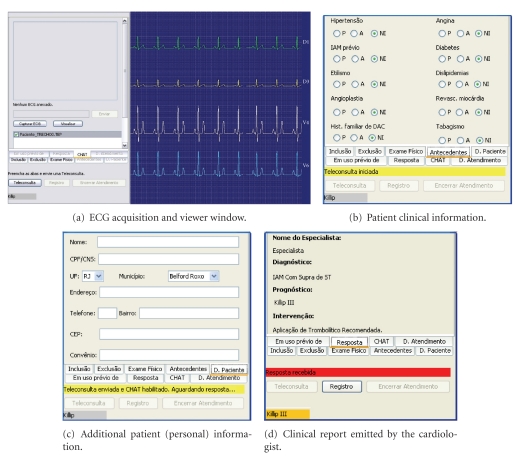
Graphical user interfaces of the paramedics' endpoint.

The graphical interface of the paramedics' endpoint comprises: (i) an ECG acquisition and viewer window (Figure 4(a), the following background) and (ii) forms to be filled during the teleconsultation (Figures 4(b) and 4(c)) as well as an interface showing the cardiologist's report (Figure 4(d)). The form shown in Figure 4(b) is used for collecting the patient clinical history which is relevant for the cardiologist's assessment, such as history for hypertension, diabetes, angioplasty, and previous AMI occurrences. It complements information of inclusion criteria (are the symptoms typical of an AMI occurrence?), and exclusion criteria (does the patient have any counterindication for thrombolysis delivery?), which are collected in two other forms (not shown in the figure). Each field in such forms is to be marked either as “P,” “A,” or “NI.” These codes stand for “Presence,” “Absence,” or “Not informed,” respectively, and have been defined by the project's medical team.  Figure 4(c) depicts an optional form used for collecting patient demographics, while Figure 4(d) presents the panel exhibiting the clinical report received from the cardiologist. This panel presents the name of the attending cardiologist, the diagnostics he/she assigns (e.g., AMI with ST elevation), the prognostics (e.g., Killip III), and recommended intervention (e.g., thrombolysis is recommended). The forms and panel are disposed as tabs which are enabled as the teleconsultation proceeds, for easing the browsability and readability in the reduced screen of a small laptop computer.

A particular detail that is still worth mentioning is that the control flow of the paramedics' application is asynchronous to both the CS and the other (opposed) endpoint (viz., the cardiologist's application). After calling the RequestWS to transmit the ECG record (Figure 4(a)), the paramedic is able to continue the collection and insertion of the patient clinical and personal information (Figures 4(b) and 4(c)). This is conducted while the application is listening to chat messages and the final clinical report from the cardiologist. In the current implementation, this listening protocol consists in a periodic checking of the web services ResponseWS and ChatWS by polling. As the final clinical report is received, the application presents on the screen a notification to the paramedic users and enables the clinical report tab (see Figure 4(d)).

### 4.4. Cardiologist's Endpoint

The cardiologist's endpoint has been implemented in Java by using the JSP (*Java Server Pages*)/Servlet technologies and the frameworks Struts and DWR (*Direct Web Remoting*). Currently, this application runs on top of the same application server as the CS although it can be hosted in another machine straightforwardly. For the webpage to be loaded, the cardiologist has selected a specific pending analysis request received from the CS. This cardiologist's application checks for such pending requests by polling the RequestWS. As the cardiologist has got access to the patient EPR, namely, ECG and clinical information, he/she can fill in the web form that molds the clinical report assigning whether or not the patient is eligible for thrombolysis. This data is published to the CS by means of the ResponseWS, which passes it to the paramedics' endpoint (see Figure 4(d)).

**Figure 5:**
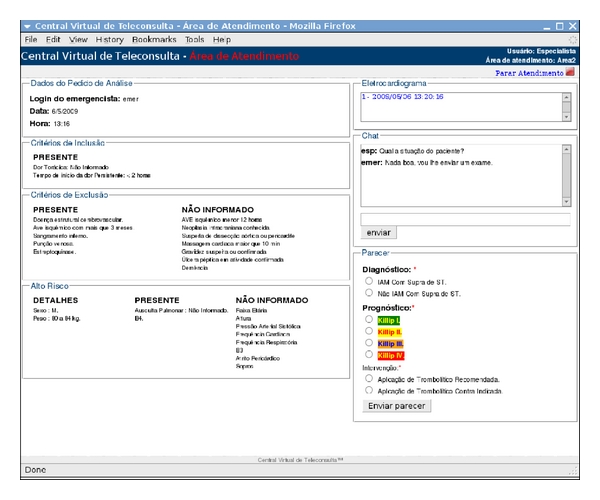
Example of webpage loaded with the patient ECG and clinical information to be assessed by the cardiologist.

Figure 5 shows the main webpage of this cardiologist's application, wherein an instance of analysis request containing an ECG and the patient clinical information is illustrated. The interface layout is divided into boxes that group different pieces of information of the analysis request received from the paramedics (i.e., the same information as described in Section 4.3). The box on the top left comprises data about the analysis request itself (paramedics ID, date and time of the request). Then, still on the left-hand side, the following boxes, from top to bottom, comprise information of inclusion criteria, exclusion criteria, and complementary clinical information provided by the paramedics. In each box, those pieces of information are grouped according to the codes “Presence,” “Absence,” and “Not Informed” which have been set by the paramedics. The boxes on the right-hand side, from top to bottom, lie in a set of links to open ECG records associated with this request, a chat interface, and finally, the form to be filled in with the cardiologist clinical report. This form has fields for diagnosis, prognostics, and recommended intervention. As the cardiologist clicks the button “Send report,” the clinical report is sent to the paramedics' endpoint.

### 4.5. Security

Security is tackled within the system, firstly, by means of standard user access and control functionality, and secondly, by providing confidentiality in the medical data exchange across the Internet. The user authentication is provided by an additional web service in the CS labeled LoginWS which is actually another functionality standing in the system functional axis (cf. Figure 2). The confidentiality during data exchange is provided by means of ciphering the SOAP messages that carry the web service requests which are filled in with the actual data. These requests are triggered by the client applications.

This confidentiality feature, as previously mentioned, is implemented by means of aspectual software components coded in both the paramedics' endpoint and the CS that intercept calls to the Sockets API and cypher them (In this implementation version, there has been no purpose in ciphering the data communication between the server side of the cardiologist's web application and the CS because they are both deployed in the same server.). The cryptography functionality in the paramedics' endpoint has been implemented by using the AspectJ language, which offers a means for implementing aspectual code in the Java platform. In the CS, it was a bit more difficult, since the JAX-WS API makes the calls to the Sockets API opaque, thus making the identification of the correct point of interception of Sockets API calls hardly possible. We have overcome this particular issue by implementing (in Java) a web micro-server hosted, again, on the same machine as the CS. This microserver is responsible for (i) intercepting the ciphered SOAP requests sent by the paramedics' endpoint; (ii) deciphering these requests; (iii) passing the deciphered requests to the proper web service; (iv) intercepting the plain SOAP responses sent by the web service; (v) ciphering these responses; and (vi) forwarding the ciphered responses to the paramedics' endpoint. 

In order to render the system working by also including this micro-server, the aspectual software component hosted in the paramedics' application has to (i) redirect the transmission of the SOAP requests to the micro-server transport address and (ii) await for it to answer with the corresponding SOAP responses. This has to be done, of course, in addition to the ciphering and deciphering of SOAP messages.

The whole process of intercepting the SOAP messages from the part of the web micro-server is transparent to the CS. Moreover, it comes about without the need to intrude with additional code in the functional components of the paramedics' application—as would be the case should we have not took advantage of the aspect-oriented programming resort.

## 5. Discussion

### 5.1. Related Work

The transmission of ECG data to a central station or a hospital dates back to 1987, when Grim et al. [23] experimented a cellular telephone transmission of 12-lead electrocardiograms from ambulance to hospital. The main objectives of such a study were testing whether the ECG signal would suffer any distortion or corruption on transmission as well as quantifying the transmission delay. From then on, different technologies have been put to test for transmitting ECG records from ambulances and emergency units in general. In a study that appeared in 2003 [24], Väisänen et al. review the compared efficiency in transmitting ECG data by using (i) conventional fax devices (as in the case of the original TIET project, cf.  Section 2.2); (ii) mobile phones; or even (iii) fax devices coupled to mobile phones. They pointed out as a result that mobile phones have been verified as efficient and reliable as traditional fax devices for the transmission of ECG data in the prehospital setting.

Along these lines, and with the rapid dissemination of mobile and wireless technologies in recent years, the ground was set for the development of a multitude of ECG transmission systems in prehospital settings (e.g., [25–28]). Such systems, however, typically focus on the remote monitoring of the patient heart and/or other vital signs in order to allow the cardiologist at a CCU or a hospital to track his/her condition and prepare for his/her arrival. Examples are the monitoring of arrhythmias in moderate-risk patients [29, 30] and the early classification of AMI patients during their transportation to a hospital with CCU [31, 32]. AToMS, in contrast, although allowing such patient remote monitoring, is meant to support prehospital real-time decision making by *teleconsultation*. As said before, it has been built with the purpose of enabling the paramedics to immediately deliver PHT (should the patient be thrombolytic eligible) as soon as the attending cardiologist has given his/her diagnosis to the emergency team in the prehospital setting.

For a general prehospital teleconsultation purpose, Kwak et al. [33] have developed a portable device that allows two-way, real-time communication between hospital doctors and emergency medical technicians. In this initiative, the data communication is carried out by means of audio and video transmission through a wireless Internet connection with an average data transmission rate of 1 Mbit/s. We highlight that such audio and video data transmission in fact requires a connection bandwidth possibly higher than that which is available in certain regions in countries like US, Brazil, and others (We are referring here to countries characterized by large territorial areas, some of which possibly lacking advanced telecommunications capability. For an overview of the current situation of US, for instance, the reader may refer to the FCC's 7th 706 report [34].). Furthermore, in the AToMS project, we have intended to transmit (i) the ECG records as files and (ii) the paramedics-cardiologist communication data as structured data. The first choice is related to the Brazilian regulations for ECG acquisition. Since we transmit the ECG files just as codified by an ANVISA-approved device, the ECG signal validation is then always warranted. The second choice, that is, to transmit structured data as opposed to audio and video data or even to use phone calls, is towards easing data storage and retrieval for the sake of auditability. Therefore, AToMS allows the interaction between the emergency and the specialized teams through an auditable system that can be used for evaluating the prescribed interventions. In sum, our project strives for making interventions in reaction to AMI cases possible in an efficient and economically advantageous way, at the location where the AMI patient receives its first treatment by an emergency team.

### 5.2. Auditability

Our concern in ensuring that the system is auditable has arisen since our first meetings with the medical team of the project. As told by them, on one side, there are still several cardiologists who are in favor of PPCI without a previous thrombolytic administration, in spite of the advantages of PHT mentioned in Section 2.1. This hinders the effective adoption of thrombolysis in case the full control of the teleconsultation session is with the cardiologist being consulted, as it is the case of the TIET project in the city of Rio de Janeiro, cf.  Section 2.2. On the other side, paramedics tend to avoid taking the responsibility of delivering thrombolysis as a consequence of the general contraindications associated to them, even though recent research demonstrates advantages of their use even in some contraindicated cases [35].

This situation imposes a practicality which inhibits the delivery of PHT to benefit more patients. AToMS, notwithstanding, is committed to be an auditable system. As previously mentioned, the coordination server records all data flows within the system (viz., EPRs, replies to consultations, and so on), building up a database that can be later used for data analysis.

### 5.3. Applicability

AToMS has a twofold applicability. The first one, and more direct, is *emergency support*, as it is the very focus of this paper. The second one, in turn, is concerned with the *syndromic surveillance* that becomes possible by relying upon the data registry recorded into the CS database.

- *Emergency Support*. This is the actual prominent applicability of the system, for which it has been designed. The first experiments with the system use have shown that it succeeds in meeting the two drawbacks of the TIET project as introduced in Section 2.2—namely, the impossibility of auditability, as well as the nonmobility of a wired facsimile infrastructure. As being location independent as long as some wireless network is available for connectivity, AToMS may be used by paramedics in ambulance services or by nonspecialized physicians in remote regions, for instance. In Section 5.6, we comment on our further perspectives in terms of the system deployment in the city of Rio de Janeiro.
- *Syndromic Surveillance*. The potential for auditability also permits us to go beyond emergency support. As the system is deployed in a second stage and gathers information on an increasing number of patient attendances, we reach syndromic surveillance as an additional but very important application. From the collected data—which is mainly structured due to the molding of the forms presented by the client applications to their users—a broad picture of the AMI patients in Rio de Janeiro is to be available. This information is rich for the sake of conducting population studies to support the release of ever more efficient public health policies.

### 5.4. Considerations on the Response Time of the System

The total response time to be experienced by the paramedics after launching a teleconsultation is mainly impacted by three different factors which prevent us from establishing a typical scenario for measuring representative response times. First, the transmission delays are typically dominated by the transmission rates available at the wireless access network. For instance, if a 3G network is available, one could expect a minimum data rate of 2 Mbps for stationary or walking users, and 384 kbps in a moving vehicle, whereas for an ordinary cellular network, the maximum transmission rate is 64 kbps. Second, the actual dominating factor for the response time of the system is essentially human based since the time taken by the cardiologist for analyzing the input data provided by the paramedic endpoint and determining the orientation for the paramedic (by sending his/her clinical report) is orders of magnitude larger than the expected latencies for data transmission. Finally, the amount of information exchanged between the paramedics and the cardiologist is both low and rather bursty (since we do not use continuous media), which only contributes for the human interaction being the actual dominating factor for the response time of the system.

### 5.5. Limitations and Future Work

We identify three main limitations in the AToMS system, which are currently the subject of ongoing work, namely,

1. as previously mentioned, two nonfunctional requirements, namely, robustness and mobility, have not been tackled yet in the AToMS system, and they gain more importance as the system gets more dependable. In [36], we proposed managing such requirements in the AToMS system at the architecture level. Now, we are working on mapping such management at the implementation level;
2. the data model we have used in formatting the ECG records as well as the patient clinical and personal data have been defined in *ad hoc* manner. We are studying the suitability of the archetype-based *openEHR* approach [37] in order to devise a data model scheme reproducible in and fit to the domain of Emergency Healthcare. This perspective has arisen from the difficulty we have faced in defining the EPR data model (ECG plus patient clinical and personal data) with the support of cardiologists. The *openEHR* standard provides a number of such data models (the so-called *archetypes*) which are conceived along several committee meetings involving physicians and informaticians;
3. we currently adopt web services as the underlying distributed software component technology. This implementation decision made us plumb many communication idioms within the system codebase—some examples are the polling and cardiologists' presence notification mechanisms. We are currently refactoring such implementation so that the system codebase becomes independent of the underlying distributed software component technology, and we are also investigating the use of XMPP (XMPP is the extensible messaging and presence protocol, a set of open technologies for instant messaging, presence, multiparty chat, voice and video calls, collaboration, lightweight middleware, content syndication, and generalized routing of XML data. More information can be found at http://xmpp.org/.) as an alternative technology of which such mechanisms as polling and presence notification are part.

As part of our ongoing work, we also envisage improving the development method we employed in the AToMS system. As with the domain-specific data model mentioned above, we are working on an architectural model scheme also straightforward to reproduce in the context of Emergency Healthcare. The idea is to come up with an entire engineering methodological apparatus prescribing a systematic workflow for a domain-specific software product line (SPL) [38]. We then expect to fetch matching patterns connecting particular architectural models fit to specific medical problems or scenarios. This methodological research pathway is an *a posteriori* realization that has emerged from our experience with AToMS.

### 5.6. Deployment and Socioeconomic Perspectives

**Figure 6:**
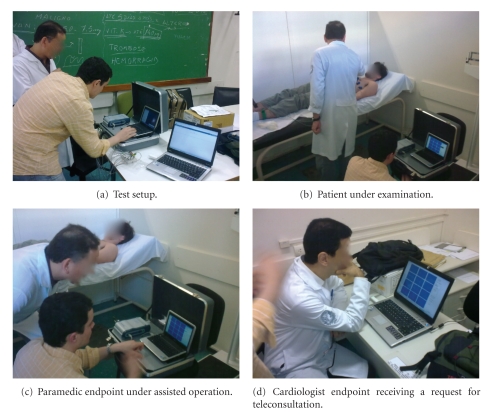
Commissioned tests. On one side, the paramedics' endpoint running on top of a highly portable laptop computer and an ECG acquisition device within a suitcase; on the other side, the cardiologist endpoint running on a web browser in a conventional notebook.

AToMS is currently in its first deployment stage in a large metropolitan area in Rio de Janeiro/Brazil. It is being integrated already in a set of ambulance emergency units of the Brazilian Mobile Emergency Healthcare System (SAMU-192) in this area, where some commissioned tests have been conducted by the medical team involved in the project (see Figure 6 for a record of one of those tests).

This research and development endeavor aims at bringing in the following socioeconomic impacts as the system is deployed:

- moving the thrombolysis delivery from the hospitals' CCUs to the emergency units, which is meant to foster a more efficient procedure in the delivery of prehospital EMS and also to speed up in-hospital management by means of the patient monitoring since his/her earlier attendance;
- increasing the rates of thrombolysis delivery (should the patient fit the eligibility criteria), possibly decreasing, hence, the rate of obits (as expected in consideration to results published in the medical literature, cf. Section 2);
- reducing the rates of surgical intervention, making possible then costs cutting in the treatment of AMI patients;
- traceability to the decision making process in the provision of care to the AMI patient—which reinforces the proper employment of the guidelines defined by public health authorities;
- setting up a statistical database for further analysis, with the purpose of identifying useful indicators to be addressed by public health policies.

## 6. Conclusions

In this paper, we have provided a comprehensive description of how the AToMS system addresses a medical problem, namely, enabling the early delivery of thrombolysis to AMI patients in the prehospital setting. We have focused on the technological aspects that had to be dealt with in order to meet the problem requirements. The system has been developed to improve the application of clinical guidelines in Rio de Janeiro by taking advantage of mobile and wireless technologies. We highlight that AToMS constitutes a complete hardware and software apparatus for meeting a medical problem that, as pointed out by the medical community (cf. Section 2), is of significant relevance.

In summary, we list below the key points of contribution brought by our experience with AToMS.

- The AToMS system is another project to provide evidence for the hypothesis that *the transmission of prehospital ECG is technically feasible and may help in reducing the system delay for ST-segment elevation acute myocardial infarction patients*.
- Mobile and wireless technologies constitute an effective means to transmit not only such ECG data, but also the patient clinical information which is relevant for the cardiologist's assessment. Furthermore, it allows the paramedics to provide additional ECGs while the patient is being displaced to a CCU, either by their own discretion (e.g., due to a sudden change in patient's condition) or in response to cardiologist's solicitations by chat.
- The design of AToMS has been also concerned with two important features for using a telemedicine system in practice: security and auditability. The potential for auditability, in particular, turns out to be a fundamental concern for the delivery of prehospital thrombolysis. As we have discussed in this paper, it encourages the medical team not to avoid responsibility in applying thrombolysis.

We conclude the paper by echoing the World Health Organization's definition for *telemedicine*, namely,

“the delivery of healthcare services, where distance is a critical factor, by healthcare professionals using information and communication technologies for the exchange of valid information for diagnosis, treatment and prevention of disease and injuries […]” [39].

Because in the AToMS project we abide by this definition, we have strived here to report the medical and socioeconomic requirements, design decisions, and implementation details of the system, pointing out the intended impacts behind its deployment as an operational medical service. Therefore, we submit that AToMS has been designed to be an effective wireless and mobile telemedicine system.
